# Bioactive content and phenolic compounds of common medlar (*Mespilus germanica* L.) and Stern’s medlar (*M. canescens* Phipps)

**DOI:** 10.1002/fsn3.2814

**Published:** 2022-03-21

**Authors:** Zeinab Sadeghinejad, Javad Erfani‐Moghadam, Ali Khadivi

**Affiliations:** ^1^ 48442 Department of Horticultural Sciences Faculty of Agriculture and Natural Resources Ilam University Ilam Iran; ^2^ 125649 Department of Horticultural Sciences Faculty of Agriculture and Natural Resources Arak University Arak Iran

**Keywords:** breeding, flavonoid content, medlar (*Mespilus*), phenolic compounds, total phenolic content

## Abstract

Medlar (*Mespilus*) is an important fruit and is rich in nutritional and medical properties. Bioactive content and phenolic compounds of leaf, fruit, and seed samples of common medlar (*M. germanica*) and Stern’s medlar (*M. canescens*) were studied. The coefficient of variation showed high values among all the characters and was more than 50.00%, ranging from 59.74 to 196.81%. The leaf of *M. canescens* showed the highest total phenolic content (14.73 mg/g dry weight, DW) and followed by 13.70 mg/g DW in the seed of *M. germanica* collected from Ilam province, Iran. The highest flavonoid content was observed in fruits of *M. germanica* collected from the north of Iran (0.90 mg/g DW), and followed by the leaf of *M. canescens* (0.53 mg/g DW). The phenolic compounds showed strong variation. Principal component analysis showed that four PCs explained 95.07% of the observed total variance. Ward dendrogram indicated two different clusters based on the characters measured, indicating high variation among the accessions. The current investigation clearly showed the potential value of the common medlar (*M. germanica*) and Stern’s medlar (*M. canescens*) germplasm, as different medlar organs are significant sources of phenolic compounds and high antioxidant activity. Therefore, these species can be considered suitable sources of natural antioxidants, and may show potential future use in food and nutraceutical supplement formulations.

## Graphical abstract

The current investigation clearly showed the potential value of the common medlar (*Mespilus germanica*) and Stern’s medlar (*M. canescens*) germplasm, as different medlar organs are significant sources of phenolic compounds and high antioxidant activity. Therefore, these species can be considered suitable sources of natural antioxidants, and may show potential future use in food and nutraceutical supplement formulations.

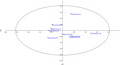

## 1 INTRODUCTION

Historically, the genus *Mespilus* has only *M. germanica* L. (common medlar), but the rare species *M. canescens* Phipps (Stern’s medlar) was identified in 1990 from a small population in the US (Phipps, 2003). Then, molecular studies showed that *M. canescens* is probably triploid, and is an intergenic hybrid between *M. germanica* and a local endemic hawthorn called *Crataegus brachyacantha* Sarg. & Engelm. or its ancestors (Lo et al., 2007). The *M. germanica* is diploid (Lo et al., 2007) and is an obscure pome fruit, especially in North America, but it is popular in Europe in middle ages. This species has a long history in medicinal and fruit applications. Its fruit is harvested in the autumn after leaf fall and stored to be soft to eat (Reich, 2004).

Different natural substances are used by humans, especially fruits, which are rich natural sources of antioxidants to prevent many diseases (Donno et al., 2017). It has been historically proven that human health is affected by the consumption of fruits, and dietary fiber is a good place in dietary guidance because they are a rich source of vitamins, minerals, and antioxidants (Slavin & Lloyd, 2012). Due to the clear role of antioxidants in health, research studies have been accelerated to find new antioxidant sources and evaluate existing antioxidant sources (Koca & Karadeniz, 2005). Also, phenols and lipids are essential for the aroma, taste, and nutritional value of the fruit. The risk of chronic diseases such as cancer as well as cardiovascular disease is reduced through their balanced consumption. Therefore, the interest in wild fruits has increased in recent years, especially since it has been proven that such fruits have high nutritional value and good therapeutic properties and new flavors (Secilmis‐Canbay et al., 2015).

Medlar fruits are edible, but they lose their edible ability a few weeks after harvest. Medlar fruits may become brown and soft on the tree or after harvest. Such fruits (over‐ripe) have sweet and slightly acidic flesh that can be eaten in this time (Lim, 2012). The fruits of medlar are climacteric and during this period, when the color of fruit is white, they cannot be eaten, because of high tannin content (Akcay et al., 2016). Some of the fruit harvested in October is stored in a cool, dark, and aerated place to soften the fruit. Another part of the fruit is used to produce pickles, which is used as an appetizer in winter (Glew et al., 2003). Medlar fruits are used to make jams, marmalades, jelly, candy, sauces, and wines. The leaves, fruits, bark, and wood of medlar are used in traditional medicine and flesh of fruit is used as a laxative (Bibalani & Sayadmahaleh, 2012; Lim, 2012).

Medlar fruits are a rich source of various sugars, organic acids, amino acids, pectins, carotene, polyphenols, minerals, and trace elements (Akcay et al., 2016; Glew et al., 2003; Lim, 2012). Bioactive compounds such as phenols and fatty acids are present in medlar fruit (Akcay et al., 2016). It has also been reported that this fruit is a rich source of natural antioxidants and can be used in the production of food and pharmaceutical formulations (Akbulut et al., 2016). Significant diversity in medlar populations in terms of fruit‐related characteristics in different regions has made it possible to select trees that have both good fruit quality and can be used in a variety of ways (Khadivi et al., 2019). Also, determination of phenolic compounds and bioactive content in different organs of this plant can help its nutritional and medicinal application (Glew et al., 2003). Medlar is well distributed in the southern and northern regions of Iran, but very few studies have been done on this plant, especially in terms of identifying phenolic compounds and bioactive content. Therefore, the aim of present study was to evaluate and determine the phenolic compounds and bioactive content in different organs of common medlar (*M. germanica*) and Stern's medlar (*M. canescens*).

## 2 MATERIAL AND METHODS

### 2.1 Plant material and evaluated properties

Bioactive content and phenolic compounds of leaf, fruit, and seed samples of common medlar (*M. germanica*) and Stern’s medlar (*M. canescens*) collected from southern and northern regions of Iran, were studied. Total phenolic content of fruit extracts was measured using the Folin–Ciocalteu reagent method with spectrophotometry (Singleton & Rossi, 1965). For determination of total flavonoid content, the method described by Grzegorczyk‐Karolak et al. (2015) was adopted. The phenolic compounds were determined according to the modified method of Rodriguez‐Delgado et al. (2001). The samples were diluted with distilled water in a ratio of 1:1, and centrifuged for 15 min at 15,000 *g*. Following initial filtration with filter paper and twice with 0.45 μm membrane filter (Millipore Millex‐HV Hydrophilic PVDF, Millipore), the supernatants were passed through HPLC. Chromatographic analysis was performed by Agilent 1100 (Agilent) HPLC system using DAD detector (Agilent) with 250 × 4.60 mm, 4 μm ODS column (HiChrom). As a mobile phase, Solvent A methanol:acetic acid:water (10:2:28) and Solvent B methanol:acetic acid:water (90:2:8) were used, with a flow rate of 1 ml/min and 20 μl injection volume for spectral measurements at 254 and 280 nm.

### 2.2 Statistical analysis

Analysis of variance (ANOVA) was performed to evaluate variation among the accessions based on the traits measured using SAS software (SAS Institute, 1990). Simple correlations between traits were determined using Pearson correlation coefficients (SPSS Inc.; Norusis, 1998). Principal component analysis (PCA) was used to investigate the relationship between accessions and determine the main traits effective in accession segregation using SPSS software. Hierarchical cluster analysis was performed using Ward's method and Euclidean coefficient using PAST software (Hammer et al., 2001). The first and second principal components (PC1/PC2) were used to create a scatter plot with PAST software.

## 3 RESULTS AND DISCUSSION

The coefficient of variation showed high values in all the characters and was more than 50.00%, ranging from 59.74 (total phenolic content) to 196.81% (ferulic). Thus, strong chemical variability was observed among the accessions. The leaf of *M. canescens* showed the highest total phenolic content (14.73 mg/g dry weight, DW) and followed by 13.70 mg/g DW in the seed of *M. germanica* collected from Ilam province, while the lowest total phenolic content was observed in fruits of *M. germanica* collected from the north of Iran (1.77 mg/g DW). Akbulut et al. (2016) reported that total phenolic content of fruits of *M. germanica* varied from 164 to 227 mg GAE/100 g.

The highest flavonoid content was observed in fruits of *M. germanica* collected from the north of Iran (0.90 mg/g DW), and followed by the leaf of *M. canescens* (0.53 mg/g DW). Flavonoids are important for antioxidant activities. The antioxidant capacity of *M. germanica* has been previously confirmed (Akbulut et al., 2016; Campanella et al., 2003; Rop et al., 2011; Serteser et al., 2008). The results imply that dietary antioxidants from medlar may provide health promoting effects to consumers (Akbulut et al., 2016).

The range of phenolic compounds was as follows: chlorogenic: 0.05–11.76 µg/g, caffeic: 0.32–20.74 µg/g, *P*‐coumaric: 0.00–8.75 µg/g, ferulic: 0.00–25.69 µg/g, rutin: 1.30–38.75 µg/g, quercetin: 0.54–2.50 µg/g, kaempferol: 0.00–2.37 µg/g, and cinnamic: 0.00–3.00 µg/g (Table 1). Akbulut et al. (2016) reported that the range of phenolic compounds of *M. germanica* samples was as follows (mg/100 g fresh fruit): chlorogenic: 8.35–11.74, rutin: 4.45–7.20, *P*‐Coumaric: 4.35–6.14, quercetin: 1.30–1.62, caffeic: 0.86–1.42, and ferulic: 0.19–0.52. Phenolic compounds contribute to fruit quality and nutritional value by modifying color, taste, aroma, and flavor, and also by providing beneficial health effects. These compounds also play a role in plant defensive mechanisms by counteracting reactive oxygen species, thus minimizing molecular damage due to microorganisms, insects, and herbivores (Vaya & Aviram, 1997).

**TABLE 1:** Statistical descriptive parameters for bioactive content and phenolic compounds used to study *M. germanica* and *M. canescens*

No.	Character	Unit	Min.	Max.	Mean	*SD*	CV (%)
1	Total phenolic content	mg/g DW	1.77	14.73	8.76	5.23	59.74
2	Flavonoid content	mg/g DW	0.00	0.90	0.35	0.31	87.24
3	Chlorogenic	µg/g	0.05	11.76	4.62	3.50	75.81
4	Caffeic	µg/g	0.32	20.74	11.82	6.21	52.54
5	*P*‐coumaric	µg/g	0.00	8.75	3.60	3.43	95.11
6	Ferulic	µg/g	0.00	25.69	4.16	8.19	196.81
7	Rutin	µg/g	1.30	38.75	13.53	12.61	93.17
8	Quercetin	µg/g	0.54	2.50	1.48	0.66	44.78
9	Kaempferol	µg/g	0.00	2.37	0.75	0.85	112.42
10	Cinnamic	µg/g	0.00	3.00	0.59	1.04	174.59

Rutin showed positive correlation with *P*‐coumaric (*r* = 0.87). Chlorogenic was positively correlated with quercetin (*r* = 0.68) and kaempferol (*r* = 0.70). Cinnamic showed positive correlation with ferulic (*r* = 0.90) (Table 2). Estimating the correlation between the properties provides useful information for breeders that they can use in designing a high‐performance design to study genotypes (Khadivi & Arab, 2021).

**TABLE 2:** Simple correlations between bioactive content and phenolic compounds in the studied *M. germanica* and *M. canescens* accessions

Character	Phenol	Flavonoid	Chlorogenic	Caffeic	*P*‐coumaric	Ferulic	Rutin	Quercetin	Kaempferol	Cinnamic
Phenol	1									
Flavonoid	0.00	1								
Chlorogenic	0.66	0.32	1							
Caffeic	0.59	0.20	0.58	1						
*P*‐coumaric	0.02	0.04	−0.31	0.57	1					
Ferulic	0.42	0.43	0.07	0.38	0.46	1				
Rutin	−0.13	−0.20	−0.31	0.53	0.87**	−0.01	1			
Quercetin	0.36	0.54	0.68*	0.65	0.21	0.26	0.10	1		
Kaempferol	0.17	0.42	0.70*	−0.02	−0.61	−0.18	−0.61	0.63	1	
Cinnamic	0.29	0.53	0.03	0.29	0.48	0.90**	0.01	0.47	0.04	1

*, **Correlation is significant at *p* ≤ 0.05 and 0.01 levels, respectively.

The PCA was performed to identify the main distinguishing characteristics of the variability. As a criterion for extracting the main components, eigenvalues >1.00 were taken to determine which of the PC scores represented the greatest value of variation. For each component, the load values above 0.65 were considered significant, which indicated four components (Table 3). The four PCs explained 95.07% of the observed total variance that 26.41% of the variance was accounted for PC1, followed by 24.25% for PC2, 23.59% for PC3, and 20.82% for PC4. *P*‐coumaric (0.91) and rutin (0.99) were found to be influential on PC1. Flavonoid (0.71), quercetin (0.87), and kaempferol (0.78) showed positive and significant correlations with PC2. The PC3 was correlated with ferulic (0.95) and cinnamic (0.94). Chlorogenic (0.73), caffeic (0.65), and total phenolic content (0.94) were found to be influential on PC4.

**TABLE 3:** Eigenvalues of the principal component axes from the PCA of bioactive content and phenolic compounds in the studied *M. germanica* and *M. canescens* accessions

Character	Component			
	1	2	3	4
Total phenolic content	−0.06	0.01	0.24	0.94**
Flavonoid content	−0.09	0.71**	0.53	−0.18
Chlorogenic	−0.21	0.62	−0.10	0.73**
Caffeic	0.63	0.36	0.14	0.65**
*P*‐coumaric	0.91**	−0.09	0.39	−0.02
Ferulic	0.12	−0.03	0.95**	0.25
Rutin	0.99**	−0.09	−0.10	−0.06
Quercetin	0.22	0.87**	0.19	0.33
Kaempferol	−0.55	0.78**	−0.13	0.17
Cinnamic	0.13	0.21	0.94**	0.07
Total	2.64	2.43	2.36	2.08
% of variance	26.41	24.25	23.59	20.82
Cumulative %	26.41	50.67	74.26	95.07

**Eigenvalues ≥0.65 are significant at the *p* ≤ 0.01 level.

**FIGURE 1:**
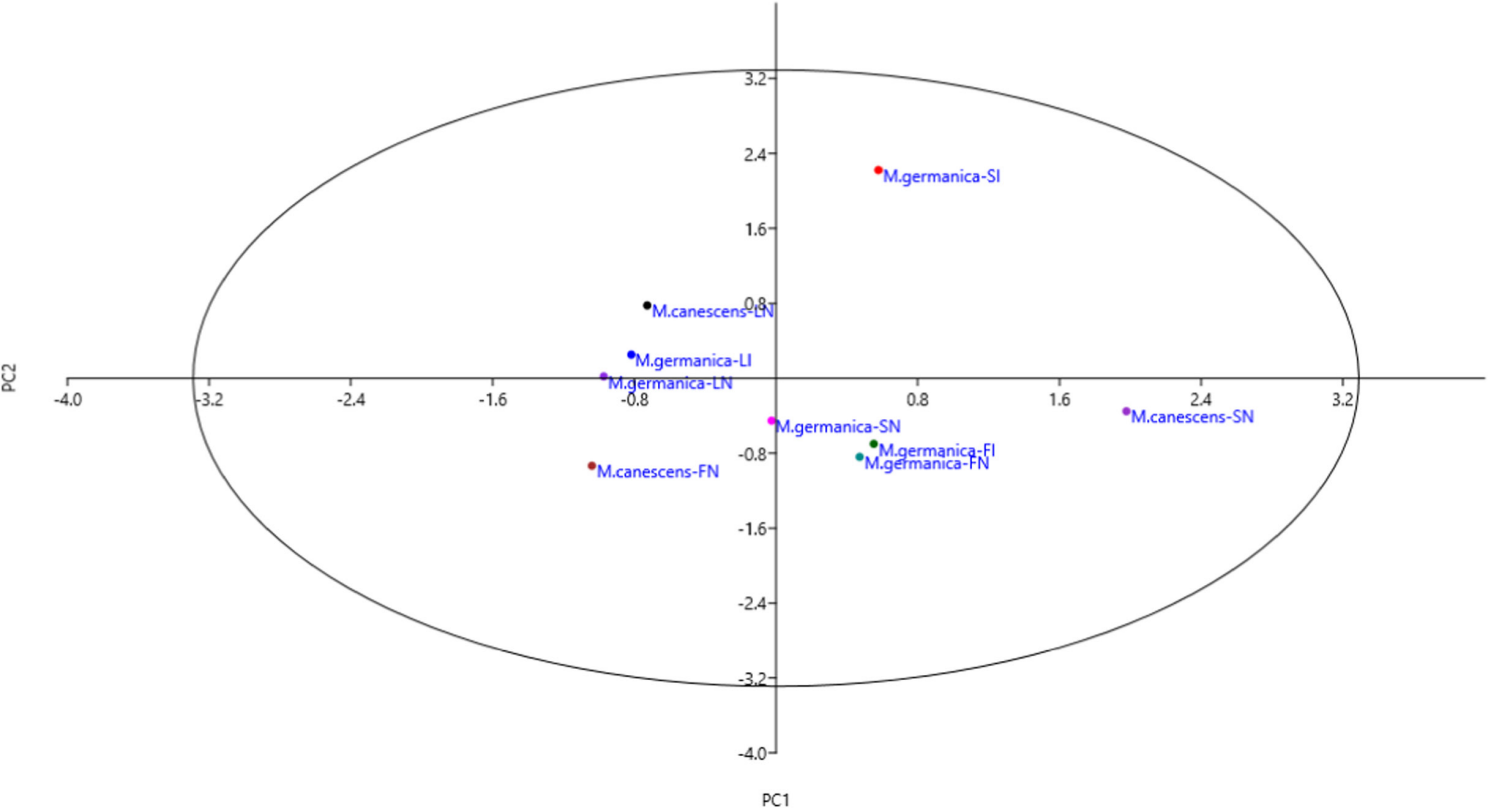
Scatter plot for the studied *M. germanica* and *M. canescens* accessions based on PC1/PC2. The symbols represent the organs and areas in the plot, including LN (leaf‐North), LI (leaf‐Ilam), FN (fruit‐North), FI (fruit‐Ilam), SN (seed‐North), and SI (seed‐Ilam)

**FIGURE 2:**
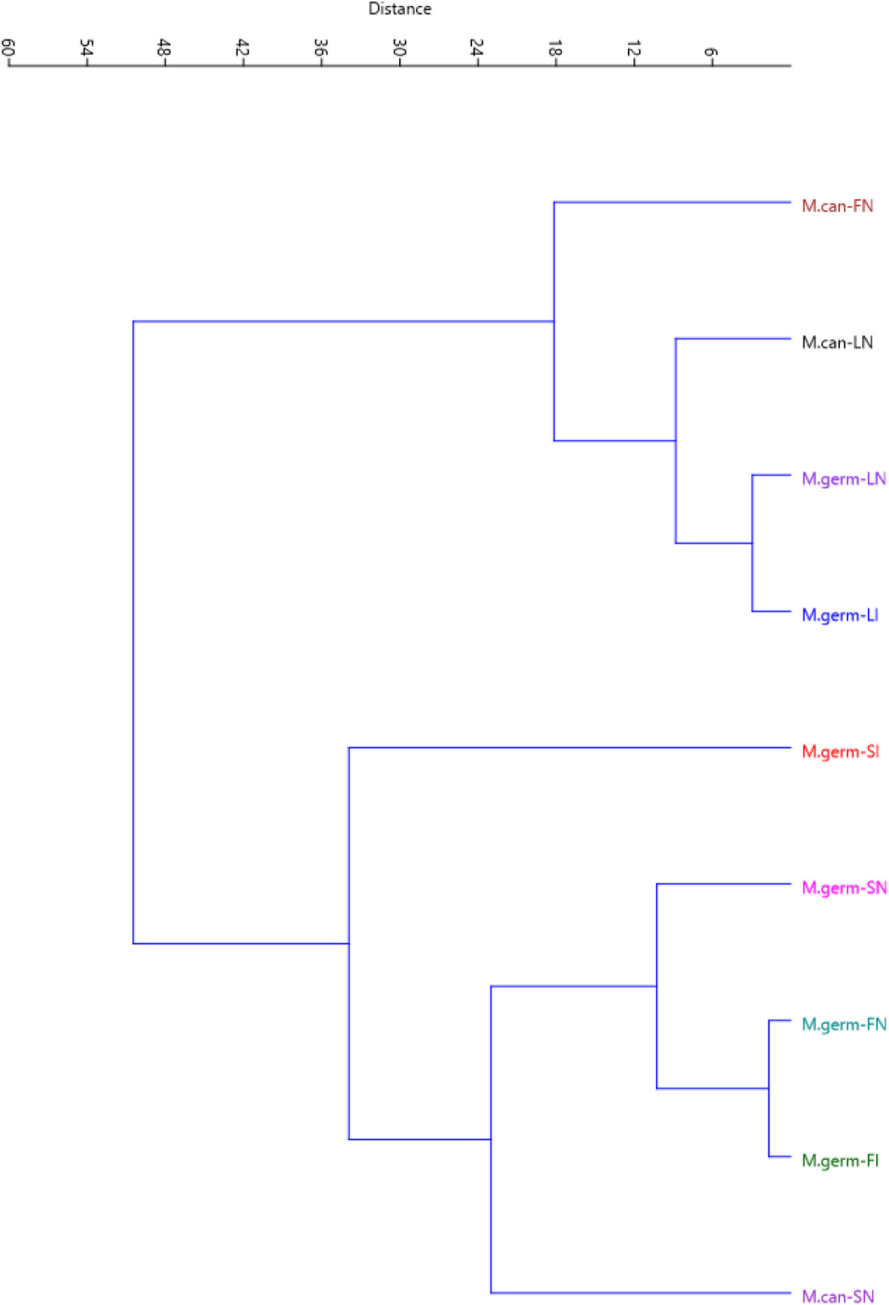
Ward cluster analysis of the studied *M. germanica* and *M. canescens* accessions based on bioactive content and phenolic compounds using Euclidean distances. The symbols represent the organs and areas in the plot, including LN (leaf‐North), LI (leaf‐Ilam), FN (fruit‐North), FI (fruit‐Ilam), SN (seed‐North), and SI (seed‐Ilam)

The projection of the studied accessions on the PC1/PC2 plot based on is presented in Figure 1. By starting from negative toward positive values of PC1, the accessions showed gradual increases in *P*‐coumaric and rutin, while starting from negative to positive values of PC2, the accessions indicated gradual increases in flavonoid, quercetin, and kaempferol. Furthermore, Ward dendrogram indicated two different clusters based on the characters measured, indicating high variation among the accessions (Figure 2).

It can be concluded that medlar germplasm studied is rich in total phenolic content. This phenomenon could be due to an induction of synthesis of antioxidant enzymes and an increase in polyphenolic concentration due to the greater exposure of the unsheltered medlar plants to extremes of temperature, and infecting/damaging organisms. Phenolic compound biosynthesis is a typical stress‐defense reaction (Ercisli et al., 2012). Rop et al. (2011) determined that total phenolic content in fruit gradually decreased after full bloom in medlar and decreased to 145 mg GAE 100 g^−1^ on the 154th day, and 93 mg GAE 100 g^−1^ on the 164th day. Selcuk and Erkan (2015) stated that total phenolic content in medlar decreased with storage time and that initial value of 763.03 mg GAE 100 g^−1^ decreased to 81.15 mg GAE 100 g^−1^ after 60 days. As can be seen from these studies, total phenolic content in fruit can vary considerably according to harvest time and time after harvest (Cevahir & Zeki‐Bostan, 2021).

## 4 CONCLUSION

The current investigation clearly showed potential value of common medlar (*M. germanica*) and Stern's medlar (*M. canescens*) germplasm, as different medlar organs are significant sources of phenolic compounds and high antioxidant activity. Therefore, these species can be considered good sources of natural antioxidants, and may show potential future use in food and nutraceutical supplement formulations. Since commercial medlar cultivars in large scale do not exist, these results could be important for determining which of these accessions to use as breeding material for future traditional breeding or advanced biotechnology studies.

## CONFLICT OF INTEREST

The authors declare no conflict of interest.

## RESEARCH INVOLVING HUMAN PARTICIPANTS AND/OR ANIMALS

None.

## INFORMED CONSENT

None.

## Data Availability

The data that support the findings of this study are available from the corresponding author upon reasonable request.
